# Sex differences in the association between chronotype and risk of depression

**DOI:** 10.1038/s41598-020-75724-z

**Published:** 2020-10-28

**Authors:** Kyung Min Kim, Seung Min Han, Kyoung Heo, Won-Joo Kim, Min Kyung Chu

**Affiliations:** 1 Department of Neurology, Yongin Severance Hospital, Yonsei University College of Medicine, Yongin, Republic of Korea; 2 Department of Neurology, Severance Hospital, Yonsei University College of Medicine, 50-1 Yonsei-ro, Seodaemun-gu, Seoul, 03722 Republic of Korea; 3 Department of Neurology, Gangnam Severance Hospital, Yonsei University College of Medicine, Seoul, Republic of Korea

**Keywords:** Neuroscience, Diseases, Medical research, Neurology

## Abstract

Information on sex differences in the association between chronotype and depression is scarce. We aimed to investigate these differences using data from the Korea National Health and Nutrition Examination Survey in 2016. Chronotypes were categorised based on mid-sleep time on free days corrected by sleep debt accumulated on workdays (MSFsc): early type, < mean MSFsc − 1 standard deviation (SD); intermediate type, between mean MSFsc − 1 SD and MSFsc + 1 SD; and late type, > mean MSFsc + 1 SD. A Patient Health Questionnaire-9 score of ≥ 10 indicated depression. Among 5550 non-shift working adults aged 19–80 years, the prevalence rates of depression in the early, intermediate, and late chronotype groups were 7.4%, 4.5%, and 9.3%, respectively. Women with late chronotype (odds ratio [OR] = 2.9, 95% confidence interval [CI] = 1.8–4.7) showed a higher risk of depression than women with intermediate chronotype after adjusting for covariates. Women with early chronotype did not show a significant difference in depression risk (OR = 1.3, 95% CI = 0.9–2.0). In conclusion, late chronotype is associated with an increased risk of depression in women but not in men. Early chronotype is not associated with depression in women or men.

## Introduction

Depression is a prevalent disease that affects 4.4% of the global population^1^. Owing to associated symptoms and comorbid conditions, depression is a significant source of disability in affected individuals and imposes a huge social burden^2^. Depression was ranked as the single largest contributor to global disability in 2015 by the World Health Organization^3^.

Chronotype refers to an individual’s circadian propensity for when to wake and be active, and when to sleep. Early chronotype (morning type, advanced sleep phase) and late chronotype (evening type, delayed sleep phase) are two extreme types of chronotypes^4^. Previous studies have reported that individuals with late chronotype are more likely to have depression or depressive symptoms^5,6^.

Sex is closely related to both depression and chronotype. Women are twice as likely to be diagnosed with depression compared to men^7^. Women experience higher rates of comorbid anxiety, a higher rate of atypical features, more somatic and cognitive-affective symptoms, and respond more favourably to selective serotonin reuptake inhibitors than men^8–10^. Prefrontal-limbic abnormalities are more prominent in women^11^. In contrast, men respond more favourably to tricyclic antidepressants than women, and prefrontal-striatal abnormalities are more marked in men^11,12^. Sex differences in chronotype have also been reported, and most studies have shown that men are more prone to show the evening chronotype or late chronotype than women^13^.

To the best of our knowledge, only one study has reported a sex difference in the association between a delayed sleep–wake schedule and depression^14^. Unfortunately, the study was conducted in young adults aged 19–25 years and did not use validated instruments to determine a delayed sleep–wake schedule. Information on the influence of sex on the association between chronotype and depression in a general population-based setting using validated instruments is currently limited.

The seventh Korea National Health and Nutrition Examination Survey (KNHANES VII) is a nationwide representative cross-sectional survey conducted by the Korea Centers for Disease Control and Prevention. Data collected during this survey may provide an opportunity to evaluate sex differences in the association between chronotype and depression. KNHANES VII, conducted in 2016, included items for depression, average sleep duration, and bedtime and wakeup time on workdays and free days. The aim of the present study was to assess whether or not there was a sex difference in the association between chronotype and depression using data from KNHANES VII.

## Results

### Participants and chronotype

**Figure 1:**
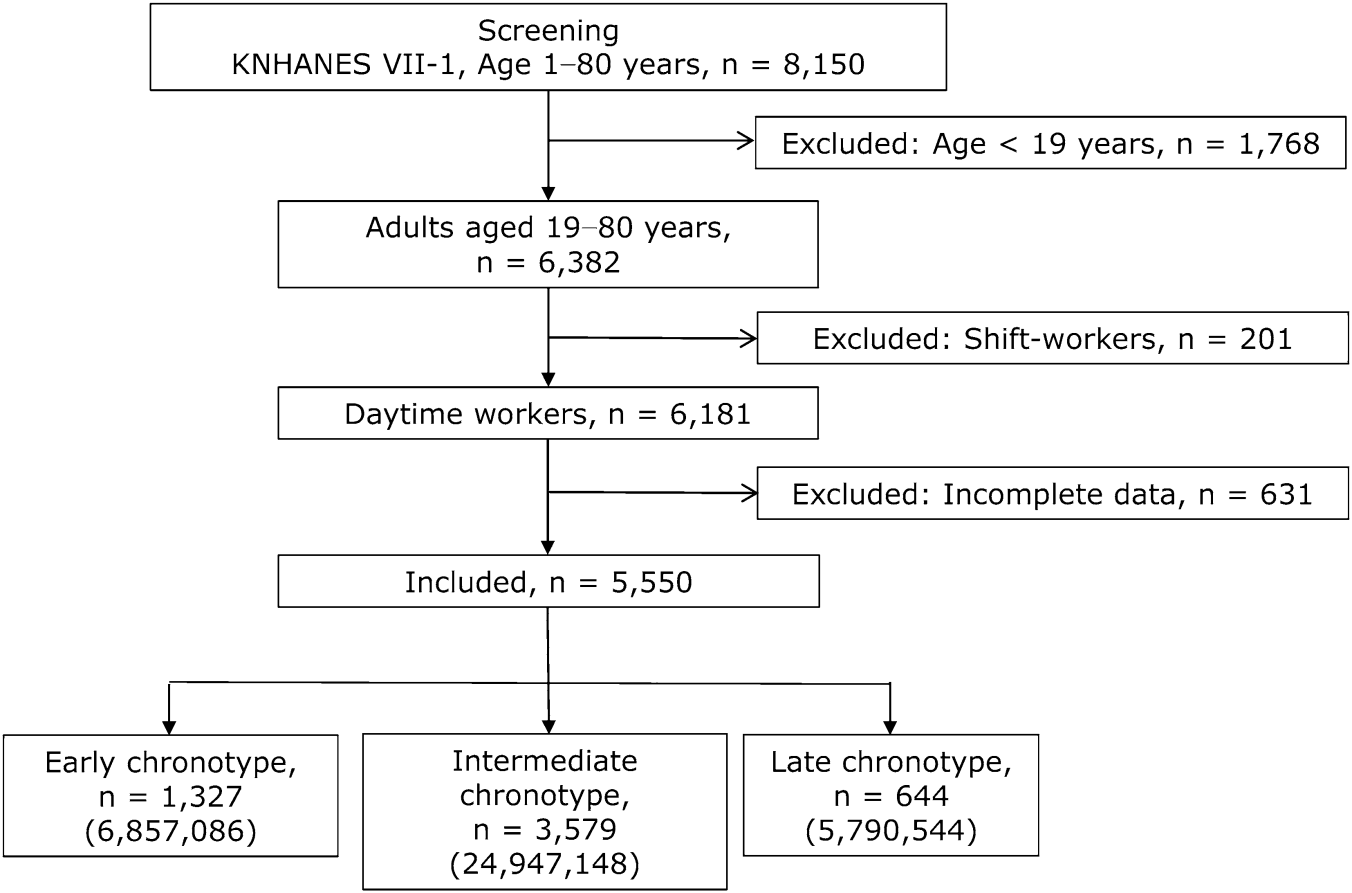
Flow chart depicting the participation of subjects in the seventh Korea National Health and Nutrition Examination Survey (KNHANES VII-1), 2016. Numbers in parentheses indicate representative numbers of the Korean population. *Shift workers were defined as those with regular day-night shift work, 24-h shift work, split-day work (≥ 2 work shifts in a day), or irregular shift work.

KNHANES VII, 2016, included data on 8,150 participants aged 1–80 years. We selected 6,382 adult participants aged 19–80 years. Among them, 201 participants were excluded because they worked in shifts, and 631 were excluded owing to incomplete data. Finally, data on 5,550 participants were used in the present study (Fig. 1). Demographic, socioeconomic, and lifestyle characteristics of the participants are summarised in Table 1.

**Table 1:** Demographic, socioeconomic, lifestyle, sleep, and chronotype characteristics of participants.

	All (n = 5,550)	Women (n = 3,222)	Men (n = 2,328)	*P*-value (women vs. men)
Age, years (mean ± SE)	46.7 ± 0.4	47.7 ± 0.5	45.7 ± 0.0	< 0.001
Chronotype, % (95% CI)				
Early type	18.2 (16.7–19.9)	17.9 (16.1–19.9)	18.5 (16.8–20.4)	0.534
Intermediate type	66.4 (64.5–68.2)	68.1 (66.0–70.1)	64.5 (61.8–67.2)	0.018
Late type	15.4 (14.0–16.9)	14.0 (12.3–15.9)	16.9 (15.0–19.0)	0.017
Sleep duration, hours (mean ± SE)				
Workday	7.0 ± 0.2	7.0 ± 0.3	7.0 ± 0.3	0.532
Free day	7.7 ± 0.3	7.7 ± 0.4	7.6 ± 0.3	0.090
Average	7.2 ± 0.2	7.2 ± 0.3	7.2 ± 0.3	0.302
MSFsc (mean ± SE)	3.5 ± 0.0 AM	3.5 ± 0.0 AM	3.6 ± 0.0 AM	0.160
Alcohol (≥ 2/week), % (95% CI)	23.8 (22.5–25.1)	12.2 (11.0–13.6)	36.1 (33.9–38.3)	< 0.001
Smoking (current), % (95% CI)	21.8 (20.2–23.5)	6.1 (5.1–7.4)	38.5 (36.0–41.1)	< 0.001
Job (yes), % (95% CI)	61.8 (59.8–63.7)	50.2 (47.8–52.7)	74.1 (71.4–76.5)	< 0.001
Education (≥ 12 yr), % (95% CI)	75.7 (73.6–77.6)	70.1 (67.6–72.5)	81.6 (79.5–83.6)	< 0.001
BMI, kg/m^2^ (mean ± SE)	24.0 ± 0.7	23.4 ± 1.0	24.6 ± 0.9	< 0.001
Depression (PHQ score ≥ 10), % (95% CI)	5.7 (4.9–6.6)	7.2 (6.1–8.5)	4.2 (3.2–5.4)	< 0.001
PHQ-9 score (mean ± SE)	2.7 ± 0.8	3.2 ± 0.1	2.1 ± 0.1	< 0.001

BMI: body mass index, CI: confidence interval, MSFsc: mid-sleep time on free days corrected by sleep debt accumulated over the workdays, PHQ-9: Patients Health Questionnaire-9, SE: standard error.

The mean mid-sleep duration on workdays, mid-sleep duration on free days (MSF), and mid-sleep duration on free days corrected by sleep debt accumulated on workdays (MSFsc) were 3.2 ± 0.0, 3.8 ± 0.0, and 3.5 ± 0.0 h, respectively. The distribution of MSFsc showed an acceptable normality (Supplementary Figure S1, skewness: 0.822, kurtosis: 3.031)^15^.

### Depression was more prevalent in women than in men

Among the 5,550 participants, 346 had Patient Health Questionnaire (PHQ)-9 scores of ≥ 10, and the prevalence of depression was calculated as 5.7% (95% confidence interval [CI] = 4.9–6.6%). The prevalence of depression in women was higher than that in men (4.2% [95% CI = 3.2–5.4%] vs. 7.2% [95% CI = 6.1–8.5%], *p* < 0.001). The mean PHQ-9 score in all participating women was higher than that in all participating men (3.2 ± 0.1 vs. 2.1 ± 0.1, *p* < 0.001). Among participants with depression, the PHQ-9 scores were not different between women and men (14.4 ± 0.3 vs. 13.9 ± 0.5 *p* = 0.224).

### Late chronotype was more prevalent in men than in women

The prevalence of late chronotype was higher in men than women (16.9% vs. 14.0%, *p* = 0.017) whereas the prevalence of intermediate chronotype was lower in men than in women (64.5% vs. 68.1%, *p* = 0.018). The prevalence of early chronotype did not significantly differ between men and women (18.5% vs. 17.9%, *p* = 0.534).

### Prevalence of depression was elevated in early and late chronotypes

**Figure 2:**
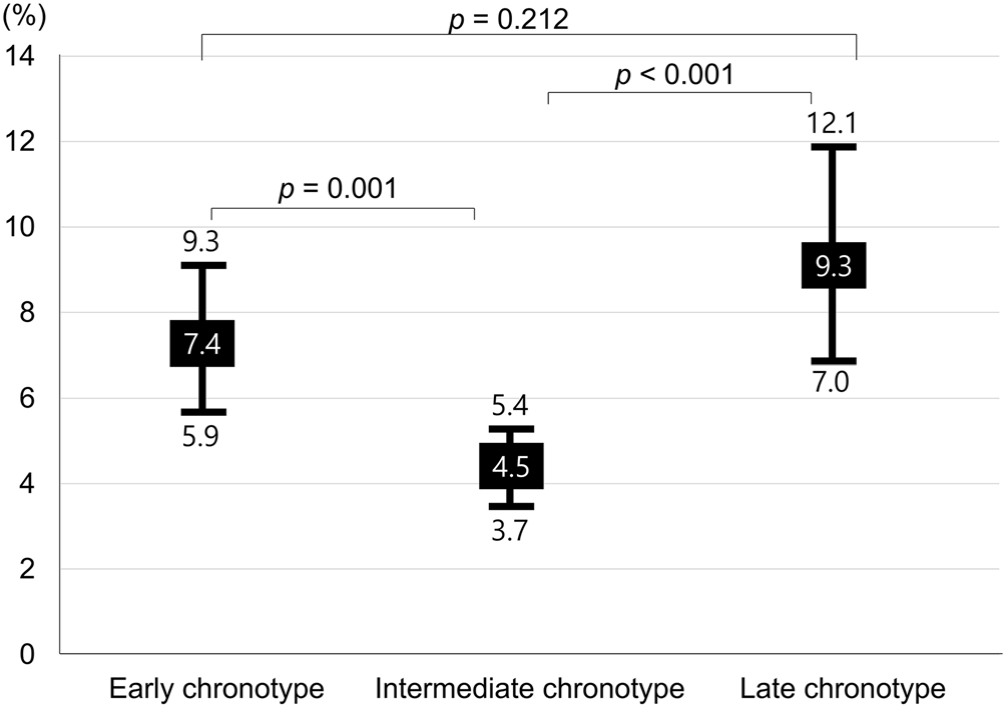
Prevalence of depression in those with early, intermediate, and late chronotypes. Mean values are presented in boxes, and 95% confidence intervals are shown below and above the bars.

The prevalence of depression was significantly different among those with early, intermediate, and late chronotypes (7.4% [95% CI = 5.9–9.3%] vs. 4.5% [95% CI = 3.7–5.4%] vs. 9.3% [95% CI = 7.0–12.1%], respectively, *p* < 0.001). Post hoc analyses revealed that there was a significant difference in the prevalence of depression between the early and intermediate chronotype groups (*p* = 0.001) and between the late and intermediate chronotype groups (*p* < 0.001). The prevalence of depression between the early and late chronotype groups was not significantly different (*p* = 0.212) (Fig. 2).

### Risk of depression was elevated in late chronotype but not in early chronotype among all participants

**Figure 3:**
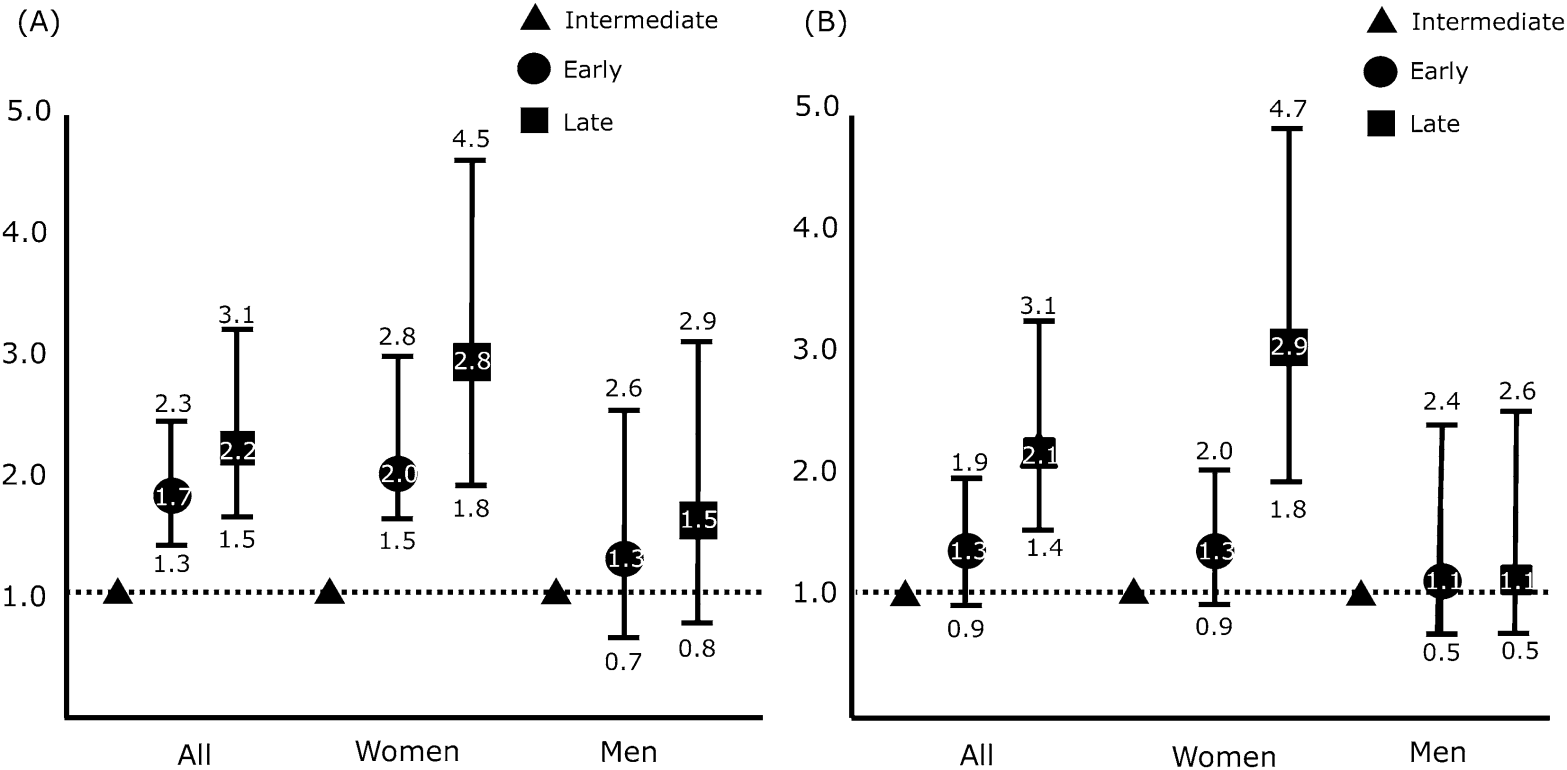
Odds ratios with 95% CI for depression in those with early (round), late (square) and intermediate (triangle) chronotypes. (**A**) Univariate ORs and (**B**) Multivariate ORs adjusted for age, sex, alcohol intake status, smoking status, job, education level, BMI, and average sleep duration. BMI: body mass index, CI: confidence interval, OR: odds ratio.

The risk of depression was significantly higher in participants with early (odds ratio [OR] = 1.7, 95% CI = 1.3–2.3) and late chronotypes (OR = 2.2, 95% CI = 1.5–3.1) than in those with intermediate chronotype, as shown by univariate analysis (Fig. 3A). After adjusting for covariates including age, body mass index (BMI), current job status, years of education, smoking status, alcohol intake, and average sleep duration, the association between depression risk and early chronotype was not significant (OR = 1.3, 95% CI = 0.9–2.0). In contrast, the association of the risk of depression with late chronotype remained significant after adjusting for covariates (OR = 2.9, 95% CI = 1.8–4.7) (Fig. 3B).

### Risk of depression was elevated in late chronotype but not in early chronotype in women

Among women, the risk of depression was higher in the early (OR = 2.0, 95% CI = 1.5–2.8) and late (OR = 2.8, 95% CI = 1.8–4.5) chronotype groups than in the intermediate chronotype group as shown by univariate analysis (Fig. 3A). After adjusting for covariates, the association of the risk of depression with late chronotype maintained its significance (OR = 2.9, 95% CI = 1.8–4.7). Nevertheless, its association with early chronotype lost significance (OR = 1.3, 95% CI = 0.9–1.9) (Fig. 3B).

### Risk of depression was not associated with chronotype in men

Among men, the risk of depression was higher in the early (OR = 1.3, 95% CI = 0.7–2.6) and late (OR = 1.5, 95% CI = 0.8–2.9) chronotype groups than in the intermediate chronotype group, but this result was not significant as shown by univariate analyses (Fig. 3A). After adjusting for covariates, the non-significant association between the risk of depression and early (OR = 1.1, 95% CI = 0.5–2.4) and late (OR = 1.1, 95% CI = 0.5–2.6) chronotypes (Fig. 3 B) persisted.

### Risk of depression according to chronotype in different age groups

We further assessed the risk of depression in participants with early and late chronotypes compared to that in those with intermediate chronotype by stratifying age into 20-year increments using multivariate logistic regression analyses adjusting for covariates (Table 2). The risk of depression was significantly elevated in all age groups among women with late chronotype. However, the risk of depression was not significantly different among age groups in women with early chronotype. In men with late chronotype, the risk of depression was not significantly different across age groups. In men with early chronotype, the risk of depression was only significantly elevated in the 40–60-year age group; no other age group showed significant differences in depression risk.

**Table 2:** Risk of depression in participants with early and late chronotypes compared to that in those with intermediate chronotype according to age groups using multivariate analysis.

Age groups, no. of corresponding participants	Intermediate chronotype, no. of depression cases/no. of corresponding participants	Early chronotype, OR and 95% CI, no. of depression cases/no. of corresponding participants	Late chronotype, OR and 95% CI, no. of depression cases/no. of corresponding participants
All participants			
Aged 19–40 years, 1,642	REF, 53/3579	1.8 (0.7–5.4), 5/52	1.9 (1.3–3.2), 42/472
Aged 41–59 years, 2,039	REF, 55/1613	2.2 (1.2–4.2), 22/276	3.1 (1.5–6.2), 12/150
Aged 60–80 years, 1,869	REF, 77/848	0.7 (0.5–1.1), 76/999	2.3 (0.8–6.7), 4/22
Women			
Aged 19–40 years, 953	REF, 36/667	3.1 (0.9–10.5), 4/26	2.1 (1.1–4.2), 30/260
Aged 41–59 years, 1,196	REF, 36/967	1.9 (0.9–4.0), 14/146	4.6 (2.0–10.7), 8/83
Aged 60–80 years, 1,073	REF, 55/502	0.8 (0.5–1.3), 57/558	3.6 (1.1–11.2), 4/13
Men			
Aged 19–40 years, 689	REF, 17/451	0.4 (0.1–4.1), 1/26	1.2 (0.5–3.2), 12/212
Aged 41–59 years, 843	REF, 19/646	3.0 (1.2–7.8), 8/130	1.5 (0.3–7.0), 4/67
Aged 60–80 years, 796	REF, 22/346	2.6 (0.9–7.7), 19/441	–, 0/9*

*No men aged 60–80 years with late chronotype had depression.

OR: odds ratio, CI: confidence internal, No.: number, REF: reference.

## Discussion

The main findings of the present study were as follows: (1) the prevalence of depression was higher in participants with early and late chronotypes than in those with intermediate chronotype; (2) depression was more prevalent in women than in men, and late chronotype was more prevalent in men than in women, and (3) after adjusting for covariates, the risk of depression was significantly associated with late chronotype in women but not in men. The association of risk of depression with early chronotype did not significantly differ in both women and men.

Biological marker studies have shown that women had a phase advance in melatonin peak time and core body temperature relative to men^16^. A meta-analysis study including 164 studies that used questionnaires such as Morningness-Eveningness Questionnaire and Composite Scale of Morningness demonstrated that men were more evening-oriented than women^13^. An American nationwide study evaluating chronotype, based on sleep onset time, wakeup time, and the Munich Chronotype Questionnaire (MCTQ), showed that men were more prone to have late chronotype than women^17^. Our study also showed similar results. This proneness to late chronotype among men observed in the present study suggests that our study properly evaluated chronotypes in women and men.

What are the potential explanations for the sex difference in the association between depression and chronotype? First, there may be a sex difference in the role of melatonin. Melatonin has a sleep-promoting effect and is a key regulatory hormone for circadian regulation, including chronotype^18,19^. Circadian abnormalities are common findings in depression, and melatonin dysregulation; including lower nocturnal melatonin levels; phase advance of melatonin onset or offset; and a delay in the peak, onset, or offset of melatonin secretion; has been reported in depression^20–23^. Furthermore, melatonin levels may also be related to alterations in serotonin and norepinephrine levels, which are important neurotransmitters in depression^24,25^. Women showed earlier onset and a higher amplitude of melatonin secretion than men^16^. Nevertheless, sex differences in melatonin dysregulation in individuals with depression and late chronotype has not been reported.

Another potential explanation is the role of sex hormones. Sex hormones can independently affect both chronotype and depression. Women with morning chronotype show earlier increases in oestradiol levels during their menstrual cycles than women with intermediate chronotype^26^. Higher testosterone levels are related to higher eveningness in adolescents^27^. Oestrogen has antianxiety and antidepressant-like effects^28^. The present study found that women with late chronotype aged 60–80 years had an increased risk of depression due to reduced levels of sex hormones. This finding indicates that sex hormones are less likely to play a major role in the sex difference. Nevertheless, sex hormones may affect the sex difference through developmental hormone exposure (organisational effects) in addition to direct effects of hormones (activational effects).

Differences in behavioural and psychological factors could be additional potential explanations. A Dutch study including 859 adults revealed that the relationships between late chronotype and depressive symptoms were mediated via sleep quality, alcohol intake, and cognitive emotion regulation strategies^29^. Another Dutch cohort study found that late chronotype (evening type) was associated with higher cognitive reactivity (depressogenic cognition)^30^. This finding suggested that depressogenic cognitions mediated the association between chronotype and depression. Women have poorer sleep quality and consume less alcohol than men^31,32^. They also exhibit more depressogenic cognitive responses to life events than men^33^. Therefore, behavioural and psychological factors may contribute to the sex difference in the association between chronotype and depression.

Since populations differ with respect to the mean and width of chronotype distribution, how ‘early’ or ‘late’ someone can be considered changes with the reference population. Therefore, there is no definitive criteria or cut-off value of MSFsc for the classification of chronotype, and various cut-off values have been proposed for establishing chronotype with the MCTQ, using 2.5%, 15%, and 20% of extreme scores of the sample^30,34,35^. In the present study, we classified early and late chronotypes as mean < MSFsc + SD and > MSFsc − SD, which corresponded approximately to the upper and lower 16% of the distribution, respectively.

The prevalence of depression in participants with early chronotype was higher than that in participants with intermediate chronotype. Nevertheless, the risk of depression in participants with early chronotype did not significantly differ from that in those with intermediate chronotype. This discrepancy might be owing to the effects of covariates. We used age, sex, BMI, average sleep duration, job status, alcohol drinking status, smoking status, and education level as covariates. Age, sex, average sleep duration, BMI, alcohol intake status, and smoking status are significantly associated with both chronotype and depression^17,36–38^. The associations of covariates with early chronotype and depression might have mitigated the significance of the association between early chronotype and depression and resulted in a non-significant association between the two conditions in the multivariate analyses. Studies on the association between early chronotype and depression have reported conflicting results^30,39–41^. Application of different covariates might be a possible reason for these conflicting outcomes.

Some limitations of the present study should be mentioned. First, we did not use the exact version of the ultrashort MCTQ (μMCTQ) in calculating MSFsc. The µMCTQ is composed of six questions regarding shift working, number of workdays per week, and sleep onset time and wakeup time on workdays and free days^42^. We calculated MSFsc based on bedtime rather than sleep onset time, and we considered the number of workdays per week as five instead of evaluating each participant’s number of workdays per week. The difference between bedtime and sleep onset time is defined as sleep latency. Although the mean sleep latency in a polysomnographic study among individuals from the general population was 18.6 ± 18.3 min, and the difference between the MSFsc based on bedtime and the actual MSFsc was not expected to be large, there was a difference between the calculated MSFsc and the actual MSFsc determined based on sleep latency^43^. Although the μMCTQ did not include a question for alarm clock use, the original version of the MCTQ did. The use of an alarm clock on free days could affect the MSFsc^44^. Nevertheless, the μMCTQ showed a good correlation with dim-light melatonin onset, a biological marker of circadian rhythm^42^. In the present study, we could not investigate alarm clock use owing to the limit on the number of items. Second, we did not evaluate the light exposure of participants. Light exposure is a key factor for determining chronotype along with age and sex^34^. The timing of light exposure plays a differential role in the circadian phase. Early light exposure advances the cycle while late light exposure delays the circadian phase^45^. Time spent outside, light dose, day length, and daily radiance were significant factors related to light exposure that showed a close association with chronotype^46^. However, items of light exposure were not included in KNHANES 2016, and we could not include light exposure in the present analyses.

Nevertheless, the present study has several strengths. First, the present study used a dataset obtained from a large sample that represented the whole population of Korea. This enabled us to properly evaluate sex difference in the association between chronotype and depression after adjusting for potential covariates. Second, we included potential covariates in the analyses including job status, years of education, smoking status, and alcohol intake, which were reported to be significantly related with chronotype and/or depression. Our analyses will provide more accurate information on the sex difference in the association between chronotype and depression.

In conclusion, we evaluated the sex difference in the association between chronotype and depression using a general population-based sample representing the whole population of Korea. We found that late chronotype was associated with an increased risk of depression in women but not in men, compared to intermediate chronotype. The risk of depression was not significantly associated with early type in women or men. These findings suggest that the influence of chronotype differs according to sex. Future research is needed to elucidate the biological mechanisms underlying the mutual effects of chronotype and depression by sex.

## Methods

### Data and participants

We used data from the KNHANES VII, which was conducted in 2016. The KNHANES is a nationwide, cross-sectional survey, representative of the entire population of Korea. The Korea Centers for Disease Control and Prevention have been conducting this survey annually for assessing the health and nutritional status of Koreans since 1998. The KNHANES adopted a stratified multistage probability sampling design to obtain a nationally representative sample of non-institutionalised civilian Koreans for data collection each year. The KNHANES collects data on a wide range of characteristics including those on sociodemographics, health, and nutrition. A detailed description of the KNHANES has been published elsewhere^47,48^. We used the data on adult participants aged 19–80 years from the KNHANES VII.

### Sleep duration

Participants’ average sleep duration was evaluated based on the response to the following two questions: ‘On average, at what time do you go to sleep and at what time do you wake up on workdays’? and ‘On average, at what time do you go to sleep and at what time do you wake up on free days’? Average sleep duration was calculated using the following formula: [(workdays sleep duration × 5) + (free days sleep duration × 2)]/7.

### Chronotype

We classified chronotype based on the μMCTQ with some modification^42^. Data on the bedtime and wakeup time on workdays and free days were used for assessing the chronotype. Chronotype was determined based on the MSFsc. MSFsc was calculated as follows: MSFsc = MSF − 0.5 × [sleep duration on free days – (5 × sleep duration on workdays + 2 × sleep duration on free days)/7]^49^. The chronotype was classified into three groups based on MSFsc: early chronotype, < mean MSFsc − 1 standard deviation (SD); intermediate chronotype, between mean MSFsc − 1 SD and MSFsc + 1 SD; and late chronotype, > mean MSFsc + 1 SD. We assumed five workdays and two free days per week for all participants. The use of an alarm clock was not considered.

### Depression

The PHQ-9 was used to assess the severity of depression^50^. A PHQ-9 score of ≥ 10 indicated depression. The Korean version of the PHQ-9 has been previously validated, and it showed 81.1% sensitivity and 89.9% specificity^51^.

### Covariates

The role of covariates was investigated to further elucidate the relationship between chronotype and depression across sex-specific groups as well as the total study population. Socioeconomic and lifestyle characteristics including BMI, current job status, years of education, smoking status, and alcohol intake have been strongly correlated with chronotype and/or depression^52–57^.

The participants’ socioeconomic characteristics and lifestyle were assessed using health interviews and examinations. Alcohol intake was classified as follows: < 2 times/week vs. ≥ 2 times/week. Smoking status was classified as follows: current vs. never or former. Current job status was also documented (employed or non-employed). Furthermore, highest achieved education level was categorised into two groups: ≥ 12 years or < 12 years. We included sex, age (continuous), job status, alcohol intake status, smoking status, average sleep duration (continuous), and BMI (continuous) as covariates in multivariate logistic regression analyses.

### Ethical approval

KNHANES VII, 2016, was a national study conducted for direct public benefit. In accordance with Article 2, Subparagraph 1 of the Bioethics and Safety Act, and Article 2, Paragraph 2, Subparagraph 1 of the Enforcement Rule of the same act, the present study did not require review by a research ethics board^58^. This study was also exempt for review by the Institutional Review Board of Severance Hospital, Yonsei University (No. 2020-2443-001). Written informed consent was obtained from all participants. This study was conducted in adherence with the KNHANES usage guidelines^59^ and the Declaration of Helsinki^60^.

### Statistical analyses

We analysed the data of KNHANES VII, using sampling weights specified in the KNHANES, which account for the complex survey design, non-response, and post-stratification, to acquire nationally representative estimates. The data in the present study are presented as weighted means or weighted proportions for continuous or categorical variables, respectively. Categorical variables are represented as numbers and percentages, and continuous variables are represented as means ± standard errors.

OR was defined as the ratio of the odds of having depression in a selected group to the odds of having depression in an unselected group. These values were compared using the chi-squared test between the three independent chronotype groups. Multivariate logistic regression analysis was used to measure the adjusted ORs and 95% CIs for depression according to sex after controlling for covariates.

The complex sample analysis module of the Statistical Package for Social Sciences version 23.0 (SPSS 23.0; IBM, Armonk, NY, USA) was used for all statistical analyses. Statistical significance was set at *p* < 0.05 two-tailed.

## Supplementary information

Supplementary Information

## Data Availability

The raw dataset used in this study is publicly available at https://knhanes.cdc.go.kr/knhanes/main.do.
