# Supplementary material for: Sex differences in the association between chronotype and risk of depression
Source: Sci Rep. 2020 Oct 28;10:18512. doi: 10.1038/s41598-020-75724-z (PMC7595163; doi:10.1038/s41598-020-75724-z)
Supplement: Supplementary file 1 — Supplementary Information [file 41598_2020_75724_MOESM1_ESM.pdf]

Sex differences in the association between chronotype and risk of depression

Kyung Min Kim, MD[1]; Seung Min Han, MD[2]; Kyoung Heo, MD, PhD[2]; Won-Joo Kim, MD, PhD[3]; Min Kyung Chu, MD, PhD[2*]

[1] Department of Neurology, Yongin Severance Hospital, Yonsei University College of Medicine, Yongin, Korea

[2] Department of Neurology, Severance Hospital, Yonsei University College of Medicine, Seoul, Korea

[3] Department of Neurology, Gangnam Severance Hospital, Yonsei University College of Medicine, Seoul, Korea

*Correspondence

Min Kyung Chu, MD, PhD,

Department of Neurology, Severance Hospital, Yonsei University College of Medicine, 50-1 Yonsei-ro, Seodaemun-gu, Seoul 03722, Republic of Korea

Telephone: +82-2-2228-1600; Fax: +82-2-393-0707;

Email: chumk@yonsei.ac.kr

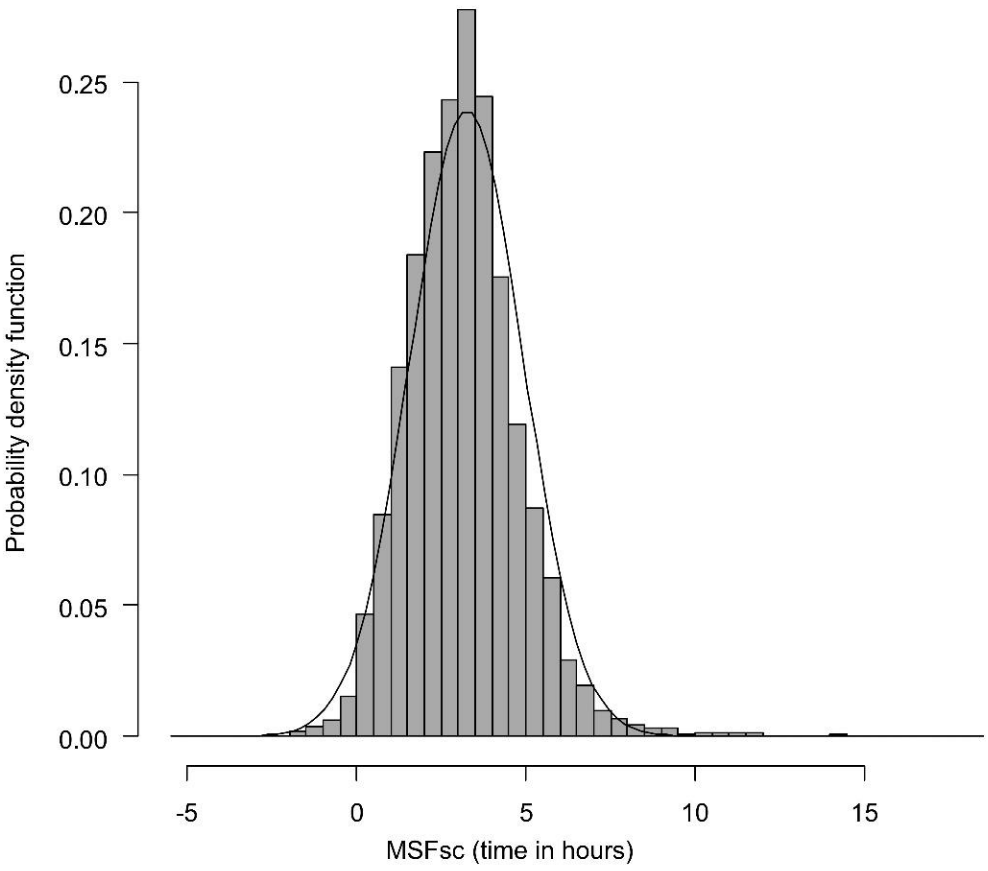

Figure S1. Distribution of MSFsc of 5500 participants

*MSFsc: mid- sleeptime onfree dayscorrected by sleep debt accumulated on workdays
